# FetalGAN: Automated Segmentation of Fetal Functional Brain MRI Using Deep Generative Adversarial Learning and Multi-Scale 3D U-Net

**DOI:** 10.3389/fnins.2022.887634

**Published:** 2022-06-07

**Authors:** Josepheen De Asis-Cruz, Dhineshvikram Krishnamurthy, Chris Jose, Kevin M. Cook, Catherine Limperopoulos

**Affiliations:** ^1^Developing Brain Institute, Department of Diagnostic Radiology, Children’s National Hospital, Washington, DC, United States; ^2^Department of Computer Science, University of Maryland, College Park, MD, United States

**Keywords:** fetal rs-fMRI, resting state, segmentation, deep learning, generative adversarial networks (GANs), 3D U-Net, fetal brain

## Abstract

An important step in the preprocessing of resting state functional magnetic resonance images (rs-fMRI) is the separation of brain from non-brain voxels. Widely used imaging tools such as FSL’s BET2 and AFNI’s 3dSkullStrip accomplish this task effectively in children and adults. In fetal functional brain imaging, however, the presence of maternal tissue around the brain coupled with the non-standard position of the fetal head limit the usefulness of these tools. Accurate brain masks are thus generated manually, a time-consuming and tedious process that slows down preprocessing of fetal rs-fMRI. Recently, deep learning-based segmentation models such as convolutional neural networks (CNNs) have been increasingly used for automated segmentation of medical images, including the fetal brain. Here, we propose a computationally efficient end-to-end generative adversarial neural network (GAN) for segmenting the fetal brain. This method, which we call FetalGAN, yielded whole brain masks that closely approximated the manually labeled ground truth. FetalGAN performed better than 3D U-Net model and BET2: FetalGAN, Dice score = 0.973 ± 0.013, precision = 0.977 ± 0.015; 3D U-Net, Dice score = 0.954 ± 0.054, precision = 0.967 ± 0.037; BET2, Dice score = 0.856 ± 0.084, precision = 0.758 ± 0.113. FetalGAN was also faster than 3D U-Net and the manual method (7.35 s vs. 10.25 s vs. ∼5 min/volume). To the best of our knowledge, this is the first successful implementation of 3D CNN with GAN on fetal fMRI brain images and represents a significant advance in fully automating processing of rs-MRI images.

## Introduction

Resting state functional MRI (rs-fMRI) is an emergent technique for interrogating *in-vivo* fetal brain function. A critical step in preparing rs-fMRI images for analyses is separating brain from non-brain voxels. In most cases, fetal brain masks are generated manually, as imaging tools that are effectively used for adult whole brain segmentation do not accurately extract the fetal brain. This suboptimal performance likely arises from the presence of surrounding maternal tissue, non-standard orientation of the fetal head, and reduced gray/white matter contrast in the fetal brain. While manual segmentation of the fetal brain provides reasonable brain masks, the process is time consuming and operator dependent. Automated processes have the potential to increase efficiency of pipelines and reproducibility of results.

A growing body of literature has demonstrated that deep learning-based segmentation outperforms traditional approaches including multi-atlas registration techniques (Huo et al., 2019; Khalili et al., 2019; Dolz et al., 2020; Zhao et al., 2022). Deep convolutional neural networks (CNN) such as U-Net have achieved remarkable success for anatomical medical image segmentation and have been shown to be versatile and effective (Ronneberger et al., 2015; Yang et al., 2018; Zhao et al., 2018; Son et al., 2020). Recently, 2D U-Net has been successfully applied to fetal resting state functional MRI data (Rutherford et al., 2021), a crucial step in automating preprocessing of fetal rs-fMRI. However, there are several limitations in using CNN-based approaches for segmentation (Ronneberger et al., 2015; Xue et al., 2018; Li et al., 2019; Rutherford et al., 2021). Although U-Nets can use skip connections to combine both low- and high-level features, there is no guarantee of spatial consistency in the final segmentation map, especially at the boundaries (Isola et al., 2017; Yang et al., 2018; Zhao et al., 2018; Dhinagar et al., 2021). To address this limitation, methods that consider spatial correlations among neighboring pixels such as conditional random field and other graph cut techniques are used as post-processing refinement (Pereira et al., 2016b; Nancy, 2019; Son et al., 2020). Utilizing pair-wise potentials, however, may cause serious boundary leakage, especially in low-contrast regions (Vijayanarasimhan and Grauman, 2010). To prevent leakage and the lack of spatial consistency, methods such as patch-based networks for training CNNs and multi-scale, multi-path CNNs with different input resolutions/network architectures have been used (Pereira et al., 2016a; Havaei et al., 2017; Kamnitsas et al., 2017; Chattopadhay et al., 2018; Xiao et al., 2020; Ghimire et al., 2021; Zhang et al., 2021; Zhu et al., 2021). However, patch-based training is computationally costly. Moreover, finding the optimal patch size that achieves superior localization accuracy is challenging. Generally, traditional CNNs have a tradeoff between achieving good localization performance/higher level of semantics (i.e., correctly classifying each voxel’s label) and crisper, more well-defined boundaries. This is a potential disadvantage specifically when applied to brain segmentation of fetal rs-fMRI, which often have low-contrast boundaries, varied voxel intensities, and features at different scales/orientations (Ronneberger et al., 2015; Xue et al., 2018; Dolz et al., 2020; Rutherford et al., 2021).

Recently, generative adversarial networks (GANs) have been shown to be a robust approach for automated medical image segmentation and to yield better, stable performance compared to state-of-the-art CNN-based models (Isola et al., 2017; Xue et al., 2018; Xun et al., 2021). Using two competing neural networks—a generator and a discriminator—GANs create exemplar images that are difficult to distinguish from real (i.e., training) images, effectively modeling any distribution of data (Gonog and Zhou, 2019). The generative network creates new examples of the data while the discriminator simultaneously evaluates these exemplars in a cyclic fashion effectively giving rise to a network that self-optimizes its error rate and converges on a model with high accuracy. Specifically, adversarial losses enforced by the discriminator network consider higher-order potentials, as opposed to the pairwise correlations utilized by voxel-wise loss functions, such as softmax. This adversarial loss serves as an adaptively learned similarity measure between the predicted segmentation label maps and the annotated ground truth that improves localization accuracy while enforcing spatial contiguity at low contrast regions, including image boundaries. Various end-to-end adversarial neural networks (e.g., SegAN) have been proposed as stable and effective frameworks for automatic segmentation (SegAN) of organs such as the brain, chest, and abdomen, among others (Frid-Adar et al., 2018; Giacomello et al., 2020; Xun et al., 2021; Zhu et al., 2021). Furthermore, a recent study by Chen et al. (2022) showed that a GAN-based paradigm improved the robustness and generalizability of deep learning models like graph neural networks (GNNs). Using their model on multi-modal MRI data, they identified autism spectrum disorders (ASD) with higher accuracy (74.7%) compared to other state-ot-the-art deep learning methods.

Motivated by SegAN, here, we propose FetalGAN, a GAN based end-to-end architecture for the automated segmentation of fetal rs-fMRI brain images. FetalGAN addresses the previously described drawbacks of deep CNNs and may be better suited for low-contrast fetal rs-fMRI. We hypothesized that FetalGAN will produce whole brain labels that closely approximate the manually created ground truth and will outperform deep CNN-based models (i.e., 3D U-Net) and the commonly used BET2 algorithm.

## Materials and Methods

### Data

We initially evaluated 75 rs-fMRI scans. Out of the 75 datasets, four were excluded from further analyses: three had image dimensions (x, y, or z) that exceeded the chosen patch size of 32 × 32 × 32, and one had incomplete demographic data. The final sample consisted of 71 datasets from 64 healthy fetuses.

Pregnant women were recruited as part of a larger study investigating brain development in healthy and high-risk fetuses. All participants had normal ultrasonograms/echocardiograms and structurally normal brains on MRI. Fetal exclusion criteria included: dysmorphic features by antenatal ultrasound, chromosomal abnormalities by amniocentesis, evidence of congenital infections, presentation after 28 weeks gestational age, and multiple gestation. Maternal exclusion criteria included: pregnant women with known psychiatric/metabolic/genetic disorders, complicated/multiple pregnancies, alcohol and/or tobacco use, maternal medications, and contraindications to MRI.

Data were collected using a 1.5T GE MRI scanner (GE Healthcare, Milwaukee, WI) with an 8-channel receiver coil. Anatomical single-shot fast spin-echo anatomical T2-weighted images were collected with the following parameters: TR = 1,100 ms, TE = 160 ms, flip angle = 90°, and slice thickness = 2 mm. Resting-state echo planar images (EPI) images were collected with the following parameters: TR = 3,000 ms, TE = 60 ms, voxel size = 2.578 mm × 2.578 mm × 3 mm, flip angle = 90°, field of view = 33 cm, matrix size = 128 × 128, and scan duration = 7 min (140 volumes). On average, 5:21 min (107 volumes) of resting-state data was available after preprocessing.

### Preprocessing

Fetal resting state data were preprocessed up to the point of brain segmentation using AFNI, unless specified otherwise (Cox, 1996). Briefly, as previously described here (De Asis-Cruz et al., 2021), fetal EPI images were slice time corrected, trimmed by removing the first four volumes to stabilize magnetic gradients, manually oriented to radiologic orientation using landmark based rigid registration (IRTK^1^), despiked, and then corrected for bias-field inhomogeneities (N4BiasFieldCorrection) (Tustison et al., 2010). At this point, the oriented EPI images were ready for motion correction. For this step, we used a two-pass registration approach optimized to correct for the high-motion typically observed in fetuses and newborns (Joshi et al., 2011; Scheinost et al., 2018). This method required two inputs: a reference volume and its mask. For each resting state (RS) dataset, a reference volume was defined using AFNI’s 3dToutcount; this identifies the volume with the lowest fraction of outlier voxels based on signal intensity trend. A brain mask was then manually drawn (JDC) for each reference brain volume using ITK-SNAP (Yushkevich et al., 2006). The goal was to automatically create this whole brain mask and provide it as input to the motion correction algorithm. The selected reference volume and the manual brain mask were utilized as inputs for training the model. During testing, the reference image was segmented using three different approaches: FSL Brain Extraction Tool v2 (BET2) (Smith, 2002), 3D U-Net (Çiçek et al., 2016), and FetalGAN. Segmentation outputs were compared to the manually created mask using the following metrics: Dice index, Jaccard score, sensitivity, specificity, and precision. We also reported the computation time for each method.

### SegAN Architecture

We used the GAN framework to automatically segment the fetal brain from rs-fMRI scans. The algorithm consisted of two neural networks: the generator (segmenter) based on 3D U-Net, and the discriminator (critic) based on a fully convolutional decoder network (Xue et al., 2018).

**FIGURE 1:**
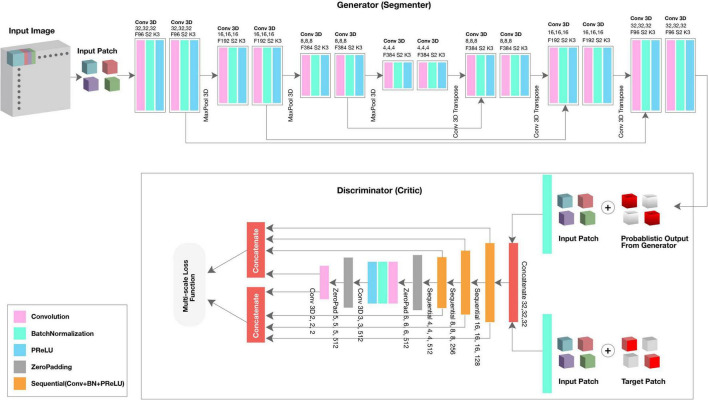
Architecture of proposed FetalGAN network.

The generator network received a 3D patch as an input and consisted of eight residual convolutional blocks with the leaky rectified linear unit (ReLU) activation, batch normalization, and maxpooling layers (Figure 1, top; see 3D U-Net Architecture for details). In the encoding branch, the upsampling layers had a kernel size of 3 × 3 × 3 with stride 2 × 2 × 2; in the decoding branch, the downsampling layers resized by a factor of 2 and used a kernel size of 2 × 2 × 2. The discriminator network’s structure was like the deconvolution block of the generator. Receiving both the ground truth and predicted label map, the discriminator extracted hierarchical features to quantify differences between these two input images. Please see Supplementary Material for a summary of generator and discriminator parameters.

SegAN learns a loss function that penalizes structural differences between the discriminator network output and target (Xue et al., 2018). Rather than computing discriminator loss for the entire network, we computed loss at each discriminator layer. The multi-scale loss function *L* was defined by Xue et al. (2018) as,


(1) $$\min_{\theta_{G}}\max_{\theta_{D}}L\,\left(\theta_{G},\theta_{D}\right)=\frac{1}{N}\,\sum_{n=1}^{N}l_{m\,a\,e}\,\left(f_{D}\,\left(x_{n}\cdot G\,\left(x_{n}\right)\right),f_{D}\,\left(x_{n}\cdot y_{n}\right)\right)$$

where *x* is the training image; *y* its corresponding ground truth; *N* is the number of training images; *l*_mae_ is the mean absolute error (MAE) or *L1* distance; *x*_*n*_⋅*G*(*x*_*n*_) is the probabilistic map generated by the generator network; *x*_*n*_⋅*y*_*n*_ is the input image masked by its corresponding ground truth; and *f*_*D*_(*x*) represent the hierarchical features extracted from image x by the discriminator network. Using a multi-scale loss function to quantify training error, the network sequentially learned both global and local features and encoded long and short-range spatial relationships between voxels. As training progressed, the generator network was able to produce probabilistic predictions that more closely approximated the expert-annotated, ground truth.

### 3D U-Net Architecture

3D U-Net, patch-based architecture was also performed (Figure 1, top). The network consisted of both an expanding and contracting path. Here, the contracting path was supplemented with successive layers where the standard pooling operators were replaced with upsampling operators to enhance image resolution. The high-resolution feature from the contracting path was then concatenated with the upsampled features from the expanding path for localization of the fetal brain. The expanding and contracting paths had four convolutional blocks, each with two Conv3D layers, BatchNormalization, and the PReLU activation function. In each convolutional block, the number of feature maps was doubled per layer (96 initial feature maps and 364 feature maps generated after the last block); a kernel size of 3 and 2 was used for the expanding and contracting paths, respectively. At the junction of the contracting/expanding path, the layers were regularized using dropout with a rate of 15%. In the expanding path, a MaxPooling (downsampling) layer with stride 2 followed each convolution block to encode the input 3D patches into feature representations at different levels. Deconvolution layers (upsampling) were used intermittently throughout the contracting path to increase the density of the sparse feature maps of the expanding path using a transpose convolution with multiple trainable filters. The successive downsampling and upsampling feature maps were concatenated to localize and learn representations after each convolution.

### Training Specifications

The SegAN was trained using a multi-scale loss function, the U-Net model using binary cross-entropy loss. For both, weights were determined using an Adam optimizer (Kingma and Ba, 2014). The models were trained on 71 reference brain volumes and their corresponding manually drawn masks. We used k-fold cross validation, where *k* = 5, for evaluating the model’s performance. Each 3D MR scan and its respective normalized mask was split into patches of size 32 × 32 × 32 with stride 2 × 2 × 2 and fed into the model for training. Given a test EPI image, we extracted the overlapped patches with size 32 × 32 × 32, and fed them to the trained network to obtain the final probability label map.

The final segmentation results were derived by averaging together the probability maps of each overlapped patch. The model training and validation are performed on NVIDIA V100 multi-GPU. After prediction, isolated and/or misidentified voxels were corrected, and internal holes were filled using morphological operations available in the openCV library (Bradski, 2000).

## Results

We evaluated 71 datasets from 64 healthy fetuses between 25 and 39.43 gestational weeks (mean GA ± SD: 33.28 ± 3.79; see Supplementary Material for age distribution). The average scan interval for the seven fetuses with two scans is 7.63 ± 2.48 weeks.

The proposed SegAN method was more time efficient than 3D U-Net, requiring, on average, 7.35 s to segment a single volume compared to 10.25 s for the latter (Table 1). BET2 was the fastest algorithm, needing only 4.40 s to extract the brain.

**TABLE 1:** Comparison of FetalGAN, 3D U-Net, and BET2.

		FetalGAN		3D U-Net				BET2		
		Mean ± SD		Mean ± SD		*p* *		Mean ± SD		*p* *
Dice		0.973 ± 0.013		0.954 ± 0.054		9.260 × 10^–4^		0.856 ± 0.084		1.124 × 10^–18^
Jaccard		0.948 ± 0.024		0.916 ± 0.082		1.993 × 10^–4^		0.756 ± 0.113		4.910 × 10^–23^
Precision		0.977 ± 0.015		0.967 ± 0.037		0.043		0.758 ± 0.113		6.685 × 10^–26^
Sensitivity		0.971 ± 0.021		0.945 ± 0.077		0.002		0.996 ± 0.011		1.493 × × 10^–17^
Specificity		0.994 ± 0.005		0.992 ± 0.010		0.239		0.915 ± 0.051		3.703 × 10^–21^
Time/patch (s)		0.05			0.08				-
Time/vol (s)		7.35			10.25				4.40

*FetalGAN compared to 3D U-Net and BET2, asterisk (*) indicates significant difference between method and FetalGAN using paired t-test.

**FIGURE 2:**
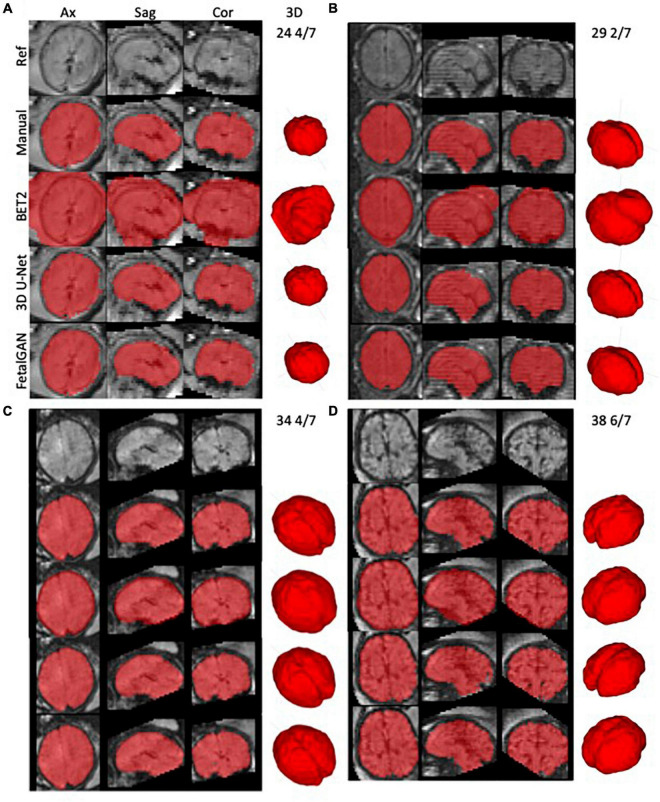
Representative whole brain masks from manual segmentation, BET2, 3D U-Net, and FetalGAN. Manual corrections were done using ITK-SNAP. FetalGAN produced the most accurate segmentation relative to the ground truth with an average Dice score of 0.942 ± 0.095. **(A)** 25 4/7 weeks, **(B)** 29 2/7 weeks, **(C)** 34 4/7 weeks, and **(D)** 38 6/7 weeks.

Whole brain segmentation outputs of FetalGAN, FSL’s BET2, and 3D U-Net were compared to the manually segmented brains. The proposed method’s Dice score, Jaccard index, precision, and specificity were significantly higher than 3d U-Net and BET2 (paired *t*-test *p* < 0.05; see Table 1). FetalGAN’s specificity was comparable to 3D U-Net and higher than BET2. Visual inspection of representative scans (Figure 2) showed FetalGAN outputs more closely resembled the ground truth. The 3D reconstructed surface of the segmentations by SegAN and U-Net is smoother when compared to the outputs of the manual and BET segmentation.

**FIGURE 3:**
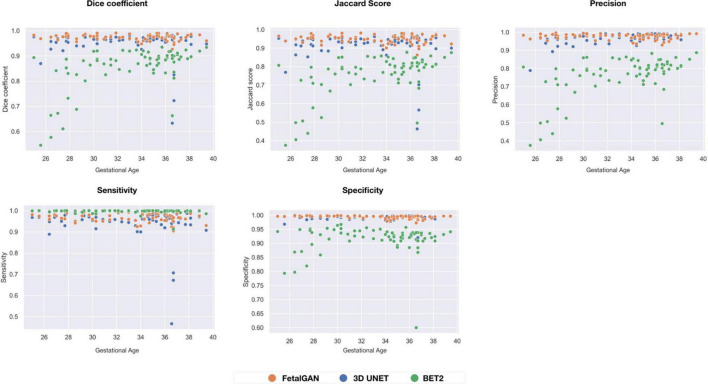
Performance scores fo FetalGAN, 3D U-Net and BET2 methods across gestation ages: **(A)** Dice coefficient, **(B)** Jaccard Score, **(C)** Precision, **(D)** Sensitivity, and **(E)** Specificity.

FetalGAN and 3D U-Net performance showed stability across GAs (Figure 3). The Dice and Jaccard scores for these two models were uncorrelated with age (Pearson *r* = –0.114, *p* = 0.230 and *r* = –0.1410, *p* = 0.241, FetalGAN and 3D U-Net, respectively; see Supplementary Table 1). FetalGAN specificity decreased with increasing GA. Despite this decrease, specificity remained high (range: 0.9723–0.9993) and was comparable to 3D U-Net and significantly better than BET2. Unlike the deep learning models, BET2 Dice coefficients and Jaccard indices were positively correlated with age (*r* = 0.558, *p* = 4.228 × 10^–7^ and *r* = 0.564, *p* = 2.985 × 10^–7^, respectively). Precision also positively scaled with increasing GA for both BET2 (*r* = 0.568, *p* = 2.396 × 10^–7^) and 3D U-Net (*r* = 0.317, *p* = 0.007).

## Discussion

We successfully implemented FetalGAN, a SegAN-based model, to accurately extract the fetal EPI brain from the maternal compartment in a sample of 71 normative fetal rs-fMRI datasets. The whole brain mask generated by FetalGAN closely approximated manually segmented images. The proposed model produced outputs superior to labels derived from 3D U-Net and FSL’s BET2. FetalGAN masks were also generated at a faster rate than U-Net and with only a minimal increase in preprocessing time compared to BET2. In addition, the proposed method produced consistently accurate segmentation across gestational ages. These findings suggest that FetalGAN is a robust, fast, and reliable approach to segmenting fetal rs-fMRI images.

To the best of our knowledge, the proposed method is the first successful application of the SegAN framework for segmentation of the fetal EPI brain and only the second automated tool for accurately separating the fetal brain from surrounding maternal tissue (Rutherford et al., 2021). We speculate that the modifications applied to the conventional GAN framework accounted for the superior performance of FetalGAN over the 3D U-Net model. Previous, conventional GAN approaches have been reported to be unstable at times due to failures during training, such as vanishing gradients and non-convergence (Isola et al., 2017; Xue et al., 2018). In other words, the adversarial loss, which classifies the image based on a scalar output by the discriminator, was unable to propagate sufficient gradients to improve the performance of the generator network (i.e., insufficient information passed on to the generator). FetalGAN utilized a multi-scale, weighted feature loss function, which effectively quantified minute differences between the generated and ground truth segmentation across multiple layers of the network. This enabled both the generator and discriminator networks to learn hierarchical features that captured relationships between voxels, especially in low contrast regions around the boundary between the fetal brain and maternal tissue. Altogether, these permitted the training process of FetalGAN to be end-to-end and stable. Moreover, FetalGAN performed faster than the comparable 3D U-Net implementation because the number of trainable parameters in the generator network was less than a 3D U-Net model. FetalGAN also outperformed BET2, likely because the boundary between fetal brain and non-brain voxels was low-contrast and BET2 relied on intensity differences between tissues to accurately estimate the boundary of the brain (Smith, 2002).

One recent study successfully implemented 2D U-Net to automatically segment the fetal EPI brain (Rutherford et al., 2021). Trained on 855 images from 129 subjects, their model yielded slightly lower performance metrics compared to FetalGAN (2D U-Net: Dice score = 0.94 ± 0.069, Jaccard index = 0.89 ± 0.069 vs. FetalGAN: Dice score = 0.973 ± 0.013, Jaccard index = 0.948 ± 0.024). In the 2D U-Net model, images were segmented in their original space; in contrast, FetalGAN was applied to oriented images. During development of our pipeline, we observed that orienting images prior to brain extraction allowed more options in subsequent preprocessing steps, thus we repositioned the brains prior to segmentation. Another critical difference between the two models is that FetalGAN was trained using 3D patches, thus it can leverage spatial information across three dimensions (i.e., interslice relationships) whereas 2D convolutional kernels obtain context only across the width and height of a slice. Moreover, with 3D U-Net, warping or normalization was not required. While we did not directly compare 2D and 3D U-Net models, previous studies have demonstrated the advantage of 3D over 2D CNNs (Nemoto et al., 2020; Woo and Lee, 2021).

FetalGAN aims to provide an automated alternative to manual segmentation of fetal rs-fMRI data. FetalGAN addresses drawbacks inherent to manual processes. First, since the process is automated, outputs are replicable. Second, the need for highly skilled operators is eliminated. Lastly, relative to manual segmentation, the time required to segment a brain volume is markedly reduced. Taken together, these three main areas of improvement are a critical step toward increasing rigor and reproducibility in fetal neuroimaging. While this is but one of the first steps in fetal rs-fMRI preprocessing, we believe that our proposed method will contribute to the field’s broader and overarching goal of creating fully automated pipelines such as what’s currently available for older children and adults with SPM,^2^ AFNI (Cox, 1996), or FSL (Jenkinson et al., 2012) (or pipelines that combine these such as fMRIPrep^3^ and CPAC,^4^ among others). The widespread availability of these tools to the larger scientific community has been instrumental in advancing our understanding of human health and disease.

Our work has several limitations. First, we used fewer training data sets for fetal EPI brain segmentation compared to a previous study (Rutherford et al., 2021). With the smaller sample size, however, we achieved comparable performance. Moreover, it should be noted that our inputs are 3D rather than 2D, thus the information that is fed into the learning model is likely comparable. Second, we used data from a single site. Additional studies that test the model on data collected from other institutions would support the generalizability of FetalGAN. Lastly, the paper demonstrated FetalGAN’s superior performance, but further studies that integrate brain extraction with other preprocessing steps to yield a fully automated pipeline are needed.

With mounting evidence supporting the fetal origins of many prevalent adult disorders including mental illness (Barker et al., 2009; Al-Haddad et al., 2019), there has been increased interest in investigating fetal functional brain development *in vivo* using MRI. FetalGAN, an implementation of SegAN for fetal rs-fMRI brain, offers a fast, automated, unbiased, and accurate alternative to currently available fetal EPI brain extraction techniques. Further improvements that focus on increasing computational efficiency, extracting the brain in the original space, and integrating FetalGAN into a fully automated fetal rs-fMRI pipeline, among others, are currently underway. It is our hope that this technique would help facilitate *in utero* investigations of emerging functional connectivity.

## Data Availability Statement

The datasets presented will be provided upon reasonable request to the corresponding author. Requests to access the datasets should be directed to CL, climpero@childrensnational.org.

## Ethics Statement

The studies involving human participants were reviewed and approved by the Institutional Review Board at Children’s National Hospital. The patients/participants provided their written informed consent to participate in this study.

## Author Contributions

JD-C: conceptualization, methodology, validation, formal analysis, investigation, data curation, writing—original draft, review, and editing, and supervision. DK and CJ: conceptualization, methodology, validation, formal analysis, investigation, data curation, writing—original draft, review, and editing, and visualization. KC: investigation and writing—original draft, review, and editing. CL: conceptualization, methodology, resources, writing—review and editing, supervision, and funding acquisition. All authors contributed to the article and approved the submitted version.

## Conflict of Interest

The authors declare that the research was conducted in the absence of any commercial or financial relationships that could be construed as a potential conflict of interest. The handling editor WZ declared a past co-authorship with CL.

## Publisher’s Note

All claims expressed in this article are solely those of the authors and do not necessarily represent those of their affiliated organizations, or those of the publisher, the editors and the reviewers. Any product that may be evaluated in this article, or claim that may be made by its manufacturer, is not guaranteed or endorsed by the publisher.

## Footnotes

1
https://github.com/BioMedIA/IRTK

2
https://www.fil.ion.ucl.ac.uk/spm/software/spm12/

3
https://fmriprep.org/en/stable/index.html

4
https://fcp-indi.github.io/docs/latest/user/quick
