# Supplementary material for: FetalGAN: Automated Segmentation of Fetal Functional Brain MRI Using Deep Generative Adversarial Learning and Multi-Scale 3D U-Net
Source: Front Neurosci. 2022 Jun 7;16:887634. doi: 10.3389/fnins.2022.887634 (PMC9209698; doi:10.3389/fnins.2022.887634)

# Supplemental Information

## Supplemental Table 1. FetalGAN summary of generator and discriminator parameters of the proposed model

**Model: "generator"**

| Layer (type) | Output Shape | Param # | Connected to |
|---|---|---|---|
| input_1 (InputLayer) | [(None, 32, 32, 32, | 0 | |
| conv3d (Conv3D) | (None, 32, 32, 32, 9 | 2688 | input_1[0][0] |
| batch_normalization (BatchNorma | (None, 32, 32, 32, 9 | 384 | conv3d[0][0] |
| p_re_lu (PReLU) | (None, 32, 32, 32, 9 | 3145728 | batch_normalization[0][0] |
| conv3d_1 (Conv3D) | (None, 32, 32, 32, 9 | 248928 | p_re_lu[0][0] |
| batch_normalization_1 (BatchNor | (None, 32, 32, 32, 9 | 384 | conv3d_1[0][0] |
| p_re_lu_1 (PReLU) | (None, 32, 32, 32, 9 | 3145728 | batch_normalization_1[0][0] |
| max_pooling3d (MaxPooling3D) | (None, 16, 16, 16, 9 | 0 | p_re_lu_1[0][0] |
| conv3d_2 (Conv3D) | (None, 16, 16, 16, 1 | 497856 | max_pooling3d[0][0] |
| batch_normalization_2 (BatchNor | (None, 16, 16, 16, 1 | 768 | conv3d_2[0][0] |
| p_re_lu_2 (PReLU) | (None, 16, 16, 16, 1 | 786432 | batch_normalization_2[0][0] |
| conv3d_3 (Conv3D) | (None, 16, 16, 16, 1 | 995520 | p_re_lu_2[0][0] |
| batch_normalization_3 (BatchNor | (None, 16, 16, 16, 1 | 768 | conv3d_3[0][0] |
| p_re_lu_3 (PReLU) | (None, 16, 16, 16, 1 | 786432 | batch_normalization_3[0][0] |
| max_pooling3d_1 (MaxPooling3D) | (None, 8, 8, 8, 192) | 0 | p_re_lu_3[0][0] |
| conv3d_4 (Conv3D) | (None, 8, 8, 8, 384) | 1991040 | max_pooling3d_1[0][0] |
| batch_normalization_4 (BatchNor | (None, 8, 8, 8, 384) | 1536 | conv3d_4[0][0] |
| p_re_lu_4 (PReLU) | (None, 8, 8, 8, 384) | 196608 | batch_normalization_4[0][0] |
| conv3d_5 (Conv3D) | (None, 8, 8, 8, 384) | 3981696 | p_re_lu_4[0][0] |
| batch_normalization_5 (BatchNor | (None, 8, 8, 8, 384) | 1536 | conv3d_5[0][0] |
| p_re_lu_5 (PReLU) | (None, 8, 8, 8, 384) | 196608 | batch_normalization_5[0][0] |
| max_pooling3d_2 (MaxPooling3D) | (None, 4, 4, 4, 384) | 0 | p_re_lu_5[0][0] |
| conv3d_6 (Conv3D) | (None, 4, 4, 4, 768) | 7963392 | max_pooling3d_2[0][0] |
| batch_normalization_6 (BatchNor | (None, 4, 4, 4, 768) | 3072 | conv3d_6[0][0] |
| p_re_lu_6 (PReLU) | (None, 4, 4, 4, 768) | 49152 | batch_normalization_6[0][0] |
| conv3d_7 (Conv3D) | (None, 4, 4, 4, 768) | 15926016 | p_re_lu_6[0][0] |
| batch_normalization_7 (BatchNor | (None, 4, 4, 4, 768) | 3072 | conv3d_7[0][0] |
| p_re_lu_7 (PReLU) | (None, 4, 4, 4, 768) | 49152 | batch_normalization_7[0][0] |
| conv3d_transpose (Conv3DTranspo | (None, 8, 8, 8, 384) | 2359680 | p_re_lu_7[0][0] |
| concatenate (Concatenate) | (None, 8, 8, 8, 768) | 0 | conv3d_transpose[0][0]<br>p_re_lu_5[0][0] |
| conv3d_8 (Conv3D) | (None, 8, 8, 8, 384) | 7963008 | concatenate[0][0] |
| batch_normalization_8 (BatchNor | (None, 8, 8, 8, 384) | 1536 | conv3d_8[0][0] |
| p_re_lu_8 (PReLU) | (None, 8, 8, 8, 384) | 196608 | batch_normalization_8[0][0] |
| conv3d_9 (Conv3D) | (None, 8, 8, 8, 384) | 3981696 | p_re_lu_8[0][0] |
| batch_normalization_9 (BatchNor | (None, 8, 8, 8, 384) | 1536 | conv3d_9[0][0] |

| | | | |
|---|---|---|---|
| p_re_lu_9 (PReLU) | (None, 8, 8, 8, 384) | 196608 | batch_normalization_9[0][0] |
| conv3d_transpose_1 (Conv3DTrans | (None, 16, 16, 16, 1 | 590016 | p_re_lu_9[0][0] |
| concatenate_1 (Concatenate) | (None, 16, 16, 16, 3 | 0 | conv3d_transpose_1[0][0]<br>p_re_lu_3[0][0] |
| conv3d_10 (Conv3D) | (None, 16, 16, 16, 1 | 1990848 | concatenate_1[0][0] |
| batch_normalization_10 (BatchNo | (None, 16, 16, 16, 1 | 768 | conv3d_10[0][0] |
| p_re_lu_10 (PReLU) | (None, 16, 16, 16, 1 | 786432 | batch_normalization_10[0][0] |
| conv3d_11 (Conv3D) | (None, 16, 16, 16, 1 | 995520 | p_re_lu_10[0][0] |
| batch_normalization_11 (BatchNo | (None, 16, 16, 16, 1 | 768 | conv3d_11[0][0] |
| p_re_lu_11 (PReLU) | (None, 16, 16, 16, 1 | 786432 | batch_normalization_11[0][0] |
| conv3d_transpose_2 (Conv3DTrans | (None, 32, 32, 32, 9 | 147552 | p_re_lu_11[0][0] |
| concatenate_2 (Concatenate) | (None, 32, 32, 32, 1 | 0 | conv3d_transpose_2[0][0]<br>p_re_lu_1[0][0] |
| conv3d_12 (Conv3D) | (None, 32, 32, 32, 9 | 497760 | concatenate_2[0][0] |
| batch_normalization_12 (BatchNo | (None, 32, 32, 32, 9 | 384 | conv3d_12[0][0] |
| p_re_lu_12 (PReLU) | (None, 32, 32, 32, 9 | 3145728 | batch_normalization_12[0][0] |
| conv3d_13 (Conv3D) | (None, 32, 32, 32, 9 | 248928 | p_re_lu_12[0][0] |
| batch_normalization_13 (BatchNo | (None, 32, 32, 32, 9 | 384 | conv3d_13[0][0] |
| p_re_lu_13 (PReLU) | (None, 32, 32, 32, 9 | 3145728 | batch_normalization_13[0][0] |
| conv3d_14 (Conv3D) | (None, 32, 32, 32, 1 | 97 | p_re_lu_13[0][0] |

Total params: 67,012,513
Trainable params: 67,004,065
Non-trainable params: 8,448

**Model: "discriminator"**

| Layer (type) | Output Shape | Param # | Connected to |
|---|---|---|---|
| input_image (InputLayer) | [(None, 32, 32, 32, | 0 | |
| target_image (InputLayer) | [(None, 32, 32, 32, | 0 | |
| concatenate_3 (Concatenate) | (None, 32, 32, 32, 2 | 0 | input_image[0][0]<br>target_image[0][0] |
| sequential (Sequential) | (None, 16, 16, 16, 1 | 449280 | concatenate_3[0][0] |
| sequential_1 (Sequential) | (None, 8, 8, 8, 256) | 2656256 | sequential[0][0] |
| sequential_2 (Sequential) | (None, 4, 4, 4, 512) | 10620928 | sequential_1[0][0] |
| zero_padding3d (ZeroPadding3D) | (None, 6, 6, 6, 512) | 0 | sequential_2[0][0] |
| conv3d_21 (Conv3D) | (None, 3, 3, 3, 512) | 16777216 | zero_padding3d[0][0] |
| batch_normalization_18 (BatchNo | (None, 3, 3, 3, 512) | 2048 | conv3d_21[0][0] |
| leaky_re_lu_6 (LeakyReLU) | (None, 3, 3, 3, 512) | 0 | batch_normalization_18[0][0] |
| zero_padding3d_1 (ZeroPadding3D | (None, 5, 5, 5, 512) | 0 | leaky_re_lu_6[0][0] |
| conv3d_22 (Conv3D) | (None, 2, 2, 2, 1) | 32769 | zero_padding3d_1[0][0] |
| flatten (Flatten) | (None, 524288) | 0 | sequential[0][0] |
| flatten_1 (Flatten) | (None, 131072) | 0 | sequential_1[0][0] |
| flatten_2 (Flatten) | (None, 32768) | 0 | sequential_2[0][0] |
| flatten_3 (Flatten) | (None, 8) | 0 | conv3d_22[0][0] |
| concatenate_4 (Concatenate) | (None, 688136) | 0 | flatten[0][0]<br>flatten_1[0][0]<br>flatten_2[0][0] |

```
                                                                              flatten_3[0][0]
==================================================================================================
Total params: 30,538,497
Trainable params: 30,534,401
Non-trainable params: 4,096
```

## Supplemental Table 2. Associations between gestational age and performance metrics

| | Dice | | Jaccard | | Precision | | Sensitivity | | Specificity | |
|---|---|---|---|---|---|---|---|---|---|---|
| | *r* | *p* | *r* | *p* | *r* | *p* | *r* | *p* | *r* | *p* |
| FetalGAN | -0.11 | 0.23 | -0.14 | 0.24 | -0.073 | 0.55 | -0.12 | 0.34 | -0.38 | 0.001 |
| 3D U-Net | -0.03 | 0.81 | -0.00 | 0.99 | 0.317 | 0.01 | -0.16 | 0.18 | 0.01 | 0.93 |
| BET2 | 0.56 | $4.23x10^{-7}$ | 0.56 | $2.99x10^{-7}$ | 0.568 | $2.40x10^{-7}$ | -0.10 | 0.39 | 0.07 | 0.55 |

* r, Pearson correlation values; significant correlations highlighted in yellow

## Supplemental Figure 1. Distribution of fetal gestational ages

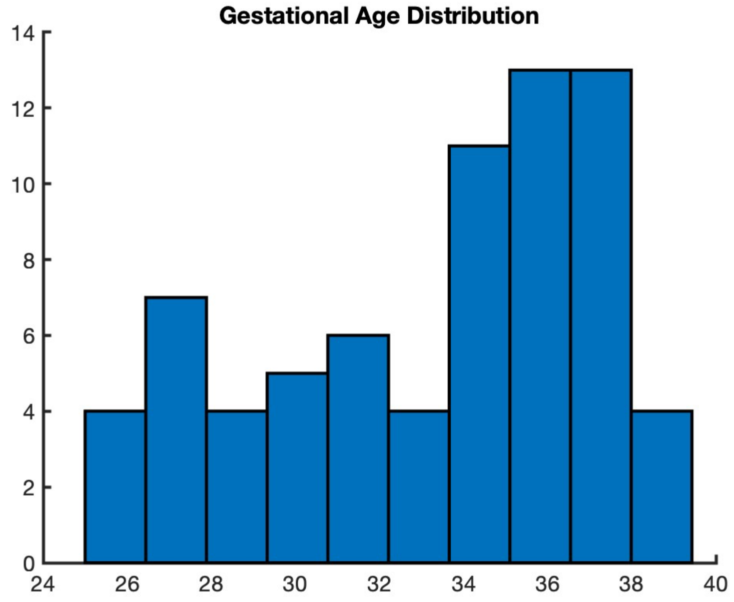

Supplement: Supplementary file 1 [file Data_Sheet_1.PDF]
